# Identification of Prophages within the *Mycobacterium avium* 104 Genome and the Link of Their Function Regarding to Environment Survival

**DOI:** 10.4236/aim.2016.613087

**Published:** 2016-11-09

**Authors:** Miao Zhao, Kerrigan Gilbert, Lia Danelishvili, Brendan Jeffrey, Luiz E. Bermudez

**Affiliations:** 1 Program of Biology, Oregon State University, Corvallis, OR, USA; 2 Department of Biomedical Sciences, College of Veterinary Medicine, Oregon State University, Corvallis, OR, USA; 3 Department of Microbiology, Oregon State University, Corvallis, OR, USA

**Keywords:** *Mycobacterum avium*, Prophage, Virulence, Environment

## Abstract

*Mycobacterium avium* is an opportunistic bacterium associated with pathogenic behavior in both humans and animals. *M. avium* has evolved as a pathogen by having an environmental component in its life style. Prophages are the integrated viral forms in bacterium genome. They constitute about 10% – 20% of genome of many bacteria and they contribute to pathogenicity of microbes. We investigated whether the *M. avium* 104 genome contained prophages and evaluated the genes/proteins for putative functions. Three prophage genes were identified in the *M. avium* 104 database, and sequences were analyzed for specific motifs. The prophage sequences were then cloned in *Mycobacterium smegmatis* and the bacterial phenotype was evaluated in gain of function assays for environmental stresses, such as tolerance to extreme temperatures, UV light, biofilm formation and resistance to acid as well as macrophage survival. The results indicate that two of the prophage genes, MAV_0696 and MAV_2265, confer *M. smegmatis* with enhanced ability to produce biofilm. Using a Real-Time PCR, it was determined that MAV_0696 and MAV_2265 transcripts were upregulated upon biofilm formation by *M. avium*. The expression of MAV_2265 gene was significantly higher at all selected time points. In addition, the expression of MAV_2265 in *M*. *smegmatis* also led to significantly greater survival rate at pH 5.0 compared to the wild-type control. None of the other physical abilities were altered by overexpressing the prophage genes in *M. smegmatis*. In summary, we identified three prophage sequences in *M. avium* 104, from which two of them were found to be associated with biofilm formation and one with resistance to the acidic environment. Future studies will identify the mechanisms involved in the prophages function.

## 1. Introduction

*Mycobacterium avium* complex (MAC) causes a broad range of opportunistic infections in humans and animals [1]. A member of the MAC known as the *Mycobacterium avium* subspecies *hominissuis* (hereafter *M. avium*) is responsible for detrimental health conditions in certain human populations, such as the elderly, children, and AIDS patients [2] [3]. *M. avium* is a highly prevalent environmental mycobacterium, with a widespread distribution in water and soil [4] [5]. Using DNA fingerprinting, *M. avium* isolates from AIDS patients were detected in residential water, indicating that *M. avium* infections might be acquired from contaminated water sources [4].

Bacteriophages (phages) infect bacterial hosts and are estimated to be the most abundant forms of life on the planet Earth [6]. While lytic phages lyse the host cells to release progeny phages, temperate phages enter a quiescent state upon viral DNA integration into the host chromosome and reproduce as prophages [7]. According to the selfish-gene concept, host-incorporated prophage genes are maintained because they contribute to the fitness of the host [8]. These genes encode for proteins capable of performing key functions, such as metabolism, adhesion, colonization, invasion, spreading, resistance to immune responses, antibiotic resistance, exotoxin production, and serum resistance [9] [10]. The roles of prophages also include increasing survival or fitness of the host by introducing new fitness factors (lysogenic conversion and transduction), genome rearrangements, gene disruption, protection from lytic infection, and destruction of competitor strains [10].

Prophages associated with host virulence have been described in a number of human pathogens, such as *Vibrio cholerae, Staphylococcus aureus, Streptococcus sp*. and others [11] [12] [13] [14]. In fact, the public database has recorded large number of prophages identified in the various bacterial genomes [7] [14] and indicates that phages represent a major driving force in the emergence and evolution of pathogenic bacteria through horizontal transfer of genes. Common examples of prophages-encoding virulence factors are diphtheria toxin, Shiga toxin, cholera toxin, type III secretion system effectors such as Salmonella-derived Sop E2.

Attempting to identify prophage sequences in the *M. avium* 104 genome, a genome-wide search of the National Center for Biotechnology Information (NCBI) database was performed. Three distinctive prophage genes MAV_0696 (hypothetical protein), MAV_2265 (putative prophage regulatory protein), and MAV_3971 (death on curing protein) were identified. Two other large regions, encompassing approximately 80 genes, have been more recently identified, but not studied in this report. The functions of these genes have yet to be defined. Therefore, biological experiments were designed to characterize *M. avium* prophage genes and to gain insight into their importance in bacterium pathogenesis.

## 2. Materials and Methods

### 2.1. Bacterial Strains and Growth Media

*M. avium* 104 is a virulent strain isolated from the blood of a patient with AIDS. *Mycobacterium smegmatis* mc^2^155 was a gift from Dr. William Jacobs Jr. (Albert Einstein College of Medicine of Yeshiva University, Bronx, NY). Both mycobacteria were grown in Middlebrook 7H9 broth (Difco Laboratories, Detroit, MI), enriched with 10% oleic acid, albumin, dextrose, and catalase (OADC, Difco) or plated on 7H10 agar (Difco) with OADC at 37°C (pH 7.2). *M. smegmatis* transformant clones were plated onto 7H10 agar containing 50 μg/ml of kanamycin (Km). *E*. *coli*strain DH5B (Stratagene, La Jolla CA) was used as the host for plasmid constructions. Luria-Bertani (LB) broth or LB agar with 50 μg of Kanamycin/ml was employed for growing *Escherichia coli* transformants. To prepare *M. avium* 104 and *M. smegmatis* inocula, bacteria grown on 7H10 agar were resuspended in Hank’s buffered salt solution (HBSS) and adjusted to McFarland Standard #2 turbidity, which corresponds to a suspension of 3.0 × 10^8^ colony forming unit (CFU) per ml. In addition, the inocula were serially diluted and plated to determine the number of CFU.

### 2.2. Bioinformatics

Identification of prophages in bacterial genome can be difficult because of the integration in the genome. Prophages harbor terminases, portal protein, head maturation protein, coat protein or still Tail tape protein. We used NCBI Gene database and PHAST [15] as the search tool to identify *M. avium* prophage sequences. *M. avium* 104 prophage genes (MAV_0696, MAV_2265, and MAV_3971) and encoding proteins were analyzed using the NCBI database (www.ncbi.nlm.nih.gov). The sequences were then matched again the data base.

### 2.3. Plasmids and PCR Settings

The promotorless mycobacterium shuttle vector pFJS3 (Table 1) was used for making the prophage gene constructs; it was propagated and purified from *E. coli* using the QIAprep Miniprep kit (QIAGEN, Valencia CA). *M. avium* strain 104 was used as a source of genomic DNA for prophage gene amplification by PCR. Three unique PCR primer sets with *Hind*III restriction site were designed to amplify the prophage genes with additional 150 bp sequence upstream of the start codon to include its native ribosomal binding site and promoter (Table 2). PCR amplification was performed in a 30 μl volume vial containing 13.5 μl of sterile H_2_O, 1 μl DMAO, 15 μl Fidelitaq mix (2X), 0.25 μl prophage-specific forward and reverse primers (100 μM). The PCR parameters were set as follows: 95°C for 5 min for the initial denaturing step, followed by 35 cycles of 95°C for 30 s, annealing at 55°C for 30 s and 72°C for 2 min, with a final extension at 72°C for an additional 5 min, and then placed at 4°C. Because MAC_3971 was not shown to have a role in any of the phenotype evaluated, we decided to use a couple variants of the promoter sequence, extended by 25 and 50 bp.

### 2.4. Construction of the Prophage Gene Clones

After PCR amplification of prophage gene sequences, the products were verified in 1% agarose gel electrophoresis, and sequenced at the Center for Gene Research and Biotechnology (CGRB), Oregon State University, Corvallis. The pFJS3 plasmid (a derivative of MV261 with the LS constitutive promoter downstream of the cloning site) was digested with Hind*III* restriction enzyme and CIP-treated for 1 h at 37°C (Table 3). The plasmid DNA and digested prophage inserts were processed for ligation, along with a positive control (digested and CIP-treated pFJS3 without any insert). The resulted positive colonies were stored in 50% glycerol a −80°C.

### 2.5. Preparation of *M. smegmatis* Competent Cells and Transformation

*M. smegmatis mc*^2^155 was obtained from glycerol stock and plated onto 7H10 agar. Bacteria were incubated at 37°C for 5 days, and then transferred into 7H9 broth enriched with OADC until OD_600_ readings of 1.0 – 2.0 A. Electro-competent cells of *M. smegmatis* were prepared as the following: the bacterial pellet was washed three times with ice-chilled sterile 10% glycerol and 0.1% Tween 80 buffer. Each time, the pellet was vortex agitated in 10 ml solution before bringing the volume to 30 ml, and centrifuged at 3500 rpm for 20 min at 4°C. The pellet obtained after the final wash was resuspended in 1 ml of the sterile 10% glycerol and placed on ice for immediate use and optimum transformation efficiency. The electroporation cuvettes were chilled at −20°C for 1 h. To each of the 200 μl of competent cells in the Eppendorf tubes, duplicates of 5 μl of purified pFJS3 or 5 μl of each transformed plasmid DNA (pFJS3 + MAV_0696, pFJS3 + MAV_2265, pFJS3 + MAV_3971) were added. These contents were then transferred to chilled cuvettes and electroporated using the parameters set on the Bio-Rad GenePulser under the following conditions: capacitance 25 μF, resistance 1000 Ω, voltage 2.5 kv. Electroporated cells were immediately recovered by gently mixing 300 μl of sterile 7H9 broth and transfered into sterile tubes for 2 h incubation at 37°C, with shaking. Each transformation was then plated onto 7H10 agar plates containing 50 μg of kanamycin/ml, and incubated for an additional 4 – 5 days. *M. smegmatis* positive clones for the three different genes were selected by PCR screening, employing the *M. avium* specific prophage primers.

To verify if the prophages genes were being expressed, five selected clones for each gene, were prepared and run in polyacrylamide gel. The observed overexpressed protein bands, using coomassie blue stain, were cut out of the gel, eluted and sent to mass spectrometry analysis at OSU Mass Spectrometry Facility, as previously reported (18). Clones in which the over expressed band correspond to the gene cloned were used in the subsequent assays.

### 2.6. Biofilm Assay

Biofilm formation was determined for *M. smegmatis* prophage clones (3 clones for each of the genes), and compared to *M. smegmatis* wild-type with or without pFJS3 vector and *M. avium* 104. Two hundred μl of 10^8^ bacteria from each clone were inoculated into polyvinyl chloride (PVC) 96-well microplates (Becton-Dickinson Labware, Franklin Lakes NJ) at room temperature in an undisturbed drawer for 3, 5, 7, 11, and 14 days. To measure the biofilm formation, the supernatants were gently removed from each well by inverting the plate onto absorbent pads. Each well was washed with 200 μl of 1× HBSS as reported [16]. The biofilms were fixed with 200 μl of methanol for 15 min with the plate lid on, followed by removal of the methanol, and incubation without the lid for an additional 15 min. Crystal violet dye can only stain the bacterial cells and not the PVC material. Fifty μl of a 1% crystal violet solution was added to each well, and the plates were incubated at room temperature for an additional 15 min. Then wells were rinsed three times with 200 μl of 1× HBSS, and 200 μl of 95% ethanol was used to dissolve the crystal violet. Biofilm formation was analyzed at 570 nm using a microplate reader (Bio-Rad, Hercules, CA).

### 2.7. Effect of Temperature

To examine the temperature effects on bacteria growth, 100 μ of 10^8^ *M. smegmatis* wild-type, prophage gene clones, and wild-type *M. avium* 104 were seeded in PCR tubes and incubated for 3 h at different temperatures (20°C, 30°C, 50°C) using a PCR temperature gradient machine. Bacterial colony counts were determined through serial dilution and plating onto 7H10 agar plates.

### 2.8. Acid Tolerance

Approximately 3.0 × 10^8^ *M. smegmatis* containing the prophage gene clones and wild- type *M. avium*104 were inoculated in HBSS at pH 2.0, pH 5.0, and pH 7.0. HBSS was adjusted to pH 2.0 and pH 5.0 with 5 M HCl and 1 M HCl, respectively, and the pH was determined using pH strips. The number of bacteria in the suspension was calculated by plating the suspension on agar plates after 2 h exposure to different pH. Acid inactivation prior to plating was carried out with NaOH.

### 2.9. Effect of UV Light

One hundred μl of 1× HBSS, containing approximately 3.0 × 10^8^ bacteria, was inoculated into PVC plastic 96-well microtiter plates and exposed to UV for different time intervals (5, 15, 30, 60 min) in the biological safety cabinet. The intensity of the UV lamp (1.d2; 40 microwatts/cm^2^) was emitted at the wavelength of 253.7 nm in the center of the work surface of the cabinet. Bacteria were placed at 25 cm from the bulb. Viable colonies from plate counts after UV exposure were compared to initial concentrations.

### 2.10 Prophage Gene Expression

To determine the expression of prophage genes upon biofilm formation, bacterial RNAs were obtained at 24h, days 3 and 5, after *M. avium* was placed in contact with a polyvinyl chloride plate surface. The Real-Time (RT) PCR was carried out using the conditions as previously described [17]. Briefly, total bacterial RNAs from broth grown bacteria (control) and from biofilms (experimental) were extracted with the combination of a guanidine thiocyanate-based buffer (Trisol) (Invitrogen, Carlsbad, CA) and rapid mechanical cell lysis of *M.avium* in a bead-beater. Prior to the real-time PCR, RNA was cleaned up with RNA clean kit (QIAGEN, Valencia, CA) and treated with DNase I. RNA quality was verified by ethidium bromide staining on the agarose gel and by OD_260/280_ nm absorption. Mycobacterial total RNA (1 μg) was reverse transcribed with 100U of Superscript II Plus RNase H^−^ Reverse Transcriptase (Invitrogen, Carlsbad, CA), using RT primers according to the manufacturer’s instruction. *M. avium* gene expressions were quantified with SYBR Green I assay by Real-Time PCR detection system using gene-specific primers.

### 2.11 Macrophage Killing Assay

It was carried out as previously reported [18]. Briefly, THP-1 macrophages were infected with *M. smegmatis* controls and *M. smegmatis* expressing the three prophage genes for 1 h. Then, the extracellular bacteria were removed by washing as described. Intracellular bacteria were quantified at 1 h, 2 days and 3 days after infection. Monolayers were lysed in presence of sterile water and 0.05% SDS for 10 min and the lysate was serially diluted and plated onto 7H10 agar plates. The number of bacteria were determined after 4 days.

### 2.12. Statistical Analysis

Data represent the means ± standard deviations from three independent experiments. The results of experimental groups and controls were compared using the Student’s *t*-test (two groups) more than two groups were confirmed by using one-way analysis of variance or ANOVA accordingly. A p < 0.05 was considered significant.

## 3. Results

### 3.1. *M. avium* Prophage Gene Analysis

**Figure 1.**
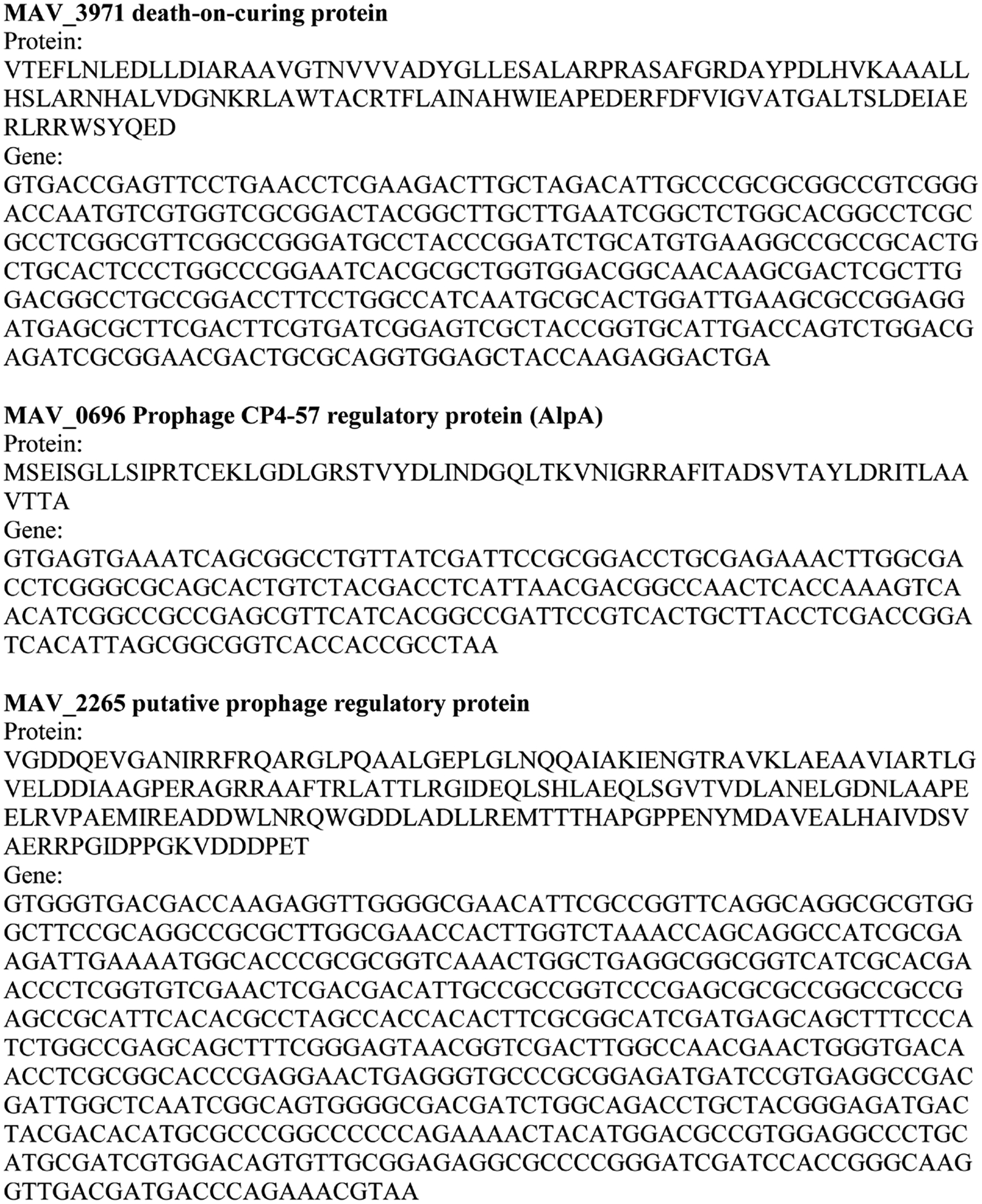
Sequences of the prophages MAV_0696, MAV_2265 and MAV_3971.

**Figure 2.**
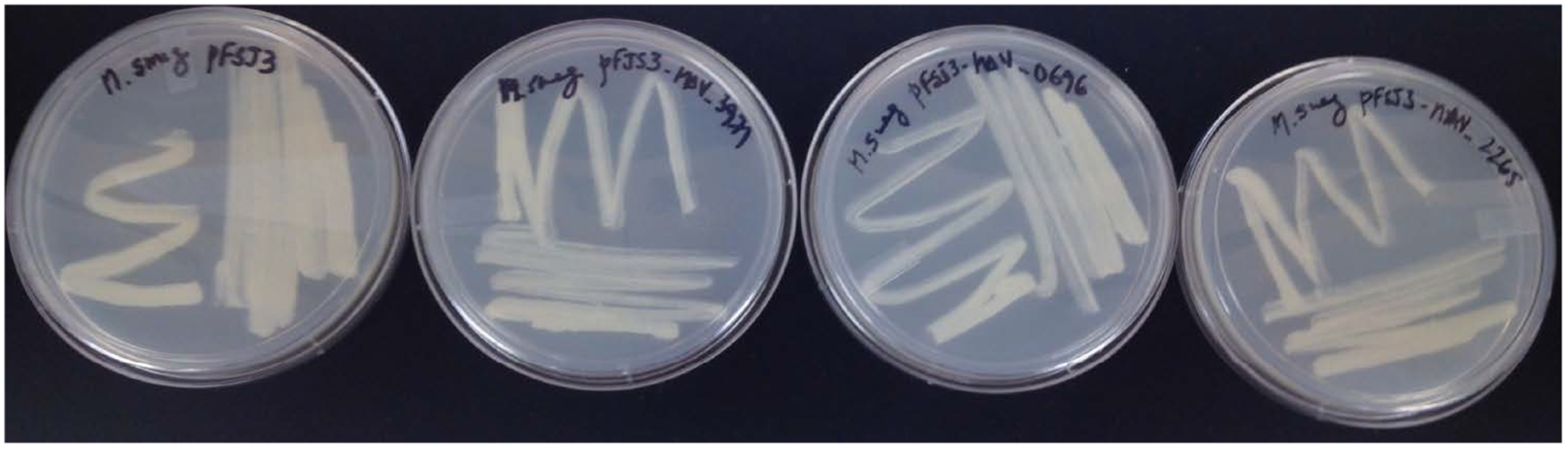
Morphotypes of *M. smegmatis* overexpressing the *M. avium* prophages MAV_0696, MAV_2265 and MAV_3971.

Figure 1 shows the genomic sequence of the prophage containing genes. G-C content, conserved domains, and putative function identified in each prophage category of *M. avium* 104 prophages are summarized in Table 2. Growth rate of selected transformants of *M. smegmatis*was examined. Three different clones of each of the *M. avium* genes in *M. smegmatis* were then incubated in presence of 7H9 broth with OADC and their growth was monitored for up to 7 days. Table 4 shows that all strains had similar growth rates. Colony morphology of the clones was also observed and the strain *M. smegmatis* with MAV_0696 has a morphotype that is drier than the WT bacterium. The other two clones were indistinguishable from the WT bacterium (Figure 2). We cloned upstream of the gene, 150 bp sequence. All the upstream sequences cloned were examined, by using bioinformatics information, for the presence of a mycobacterial promoter sequence.

### 3.2. Biofilm Assay

To determine whether the prophages genes would have a role in biofilm formation, a common characteristic of mycobacteria in the environment, we seeded *M. smegmatis* wild-type and selected three clones overexpressing the prophage proteins in HBSS and compared for differences in the biofilm robustness over time. All three selected clones for each prophage gene showed similar results. We then chose one representative clone for each prophage, for which results are shown. As shown in Table 5, expression of the prophages MAV_2265 and MAV_0696 in *M. smegmatis* resulted in greater biofilm formation at each indicated time interval when compared to both wild-type and pFJS3 containing *M. smegmatis* control strains.

### 3.3. Effects of Temperature on *M. smegmatis* Survival

Since mycobacteria are exposed to a range of different temperatures in the environment, *M. smegmatis* expressing prophages genes and wild-type strain were incubated at

#### MAV_3971 death-on-curing protein

##### Protein:

VTEFLNLEDLLDIARAAVGTNVVVADYGLLESALARPRASAFGRDAYPDLHVKAAALLHSLARNHALVDGNKRLAWTACRTFLAINAHWIEAPEDERFDFVIGVATGALTSLDEIAERLRRWSYQED

##### Gene:

GTGACCGAGTTCCTGAACCTCGAAGACTTGCTAGACATTGCCCGCGCGGCCGTCGGGACCAATGTCGTGGTCGCGGACTACGGCTTGCTTGAATCGGCTCTGGCACGGCCTCGCGCCTCGGCGTTCGGCCGGGATGCCTACCCGGATCTGCATGTGAAGGCCGCCGCACTGCTGCACTCCCTGGCCCGGAATCACGCGCTGGTGGACGGCAACAAGCGACTCGCTTGGACGGCCTGCCGGACCTTCCTGGCCATCAATGCGCACTGGATTGAAGCGCCGGAGGATGAGCGCTTCGACTTCGTGATCGGAGTCGCTACCGGTGCATTGACCAGTCTGGACGAGATCGCGGAACGACTGCGCAGGTGGAGCTACCAAGAGGACTGA

#### MAV_0696 Prophage CP4–57 regulatory protein (AlpA)

##### Protein:

MSEISGLLSIPRTCEKLGDLGRSTVYDLINDGQLTKVNIGRRAFITADSVTAYLDRITLAAVTTA

##### Gene:

GTGAGTGAAATCAGCGGCCTGTTATCGATTCCGCGGACCTGCGAGAAACTTGGCGA CCTCGGGCGCAGCACTGTCTACGACCTCATTAACGACGGCCAACTCACCAAAGTCA ACATCGGCCGCCGAGCGTTCATCACGGCCGATTCCGTCACTGCTTACCTCGACCGGA TCAC ATTAGCGGCGGTCACCACCGCCTAA

#### MAV_2265 putative prophage regulatory protein

##### Protein:

VGDDQEVGANIRRFRQARGLPQAALGEPLGLNQQAIAKIENGTRAVKLAEAAVIARTLGVELDDIAAGPERAGRRAAFTRLATTLRGIDEQLSHLAEQLSGVTVDLANELGDNLAAPEELRVPAEMIREADDWLNRQWGDDLADLLREMTTTHAPGPPENYMDAVEALHAIVDSVAERRPGIDPPGKVDDDPET

##### Gene:

GTGGGTGACGACCAAGAGGTTGGGGCGAACATTCGCCGGTTCAGGCAGGCGCGTGGGCTTCCGCAGGCCGCGCTTGGCGAACCACTTGGTCTAAACCAGCAGGCCATCGCGAAGATTGAAAATGGCACCCGCGCGGTCAAACTGGCTGAGGCGGCGGTCATCGCACGAACCCTCGGTGTCGAACTCGACGACATTGCCGCCGGTCCCGAGCGCGCCGGCCGCCGAGCCGCATTCACACGCCTAGCCACCACACTTCGCGGCATCGATGAGCAGCTTTCCCATCTGGCCGAGCAGCTTTCGGGAGTAACGGTCGACTTGGCCAACGAACTGGGTGACAACCTCGCGGCACCCGAGGAACTGAGGGTGCCCGCGGAGATGATCCGTGAGGCCGACGATTGGCTCAATCGGCAGTGGGGCGACGATCTGGCAGACCTGCTACGGGAGATGACTACGACACATGCGCCCGGCCCCCCAGAAAACTACATGGACGCCGTGGAGGCCCTGCATGCGATCGTGGACAGTGTTGCGGAGAGGCGCCCCGGGATCGATCCACCGGGCAAGGTTGACGATGACCCAGAAACGTAA

different temperatures (20°C, 37°C, 50°C) to establish whether the prophages genes confer advantage to the bacterium to survive in extreme temperatures. In all three temperatures tested, the presence of prophages genes did not offer any significant advantage to *M. smegmatis* clones.

### 3.4. Effects of Acidic Environment on *M. smegmatis* Survival

**Figure 3.**
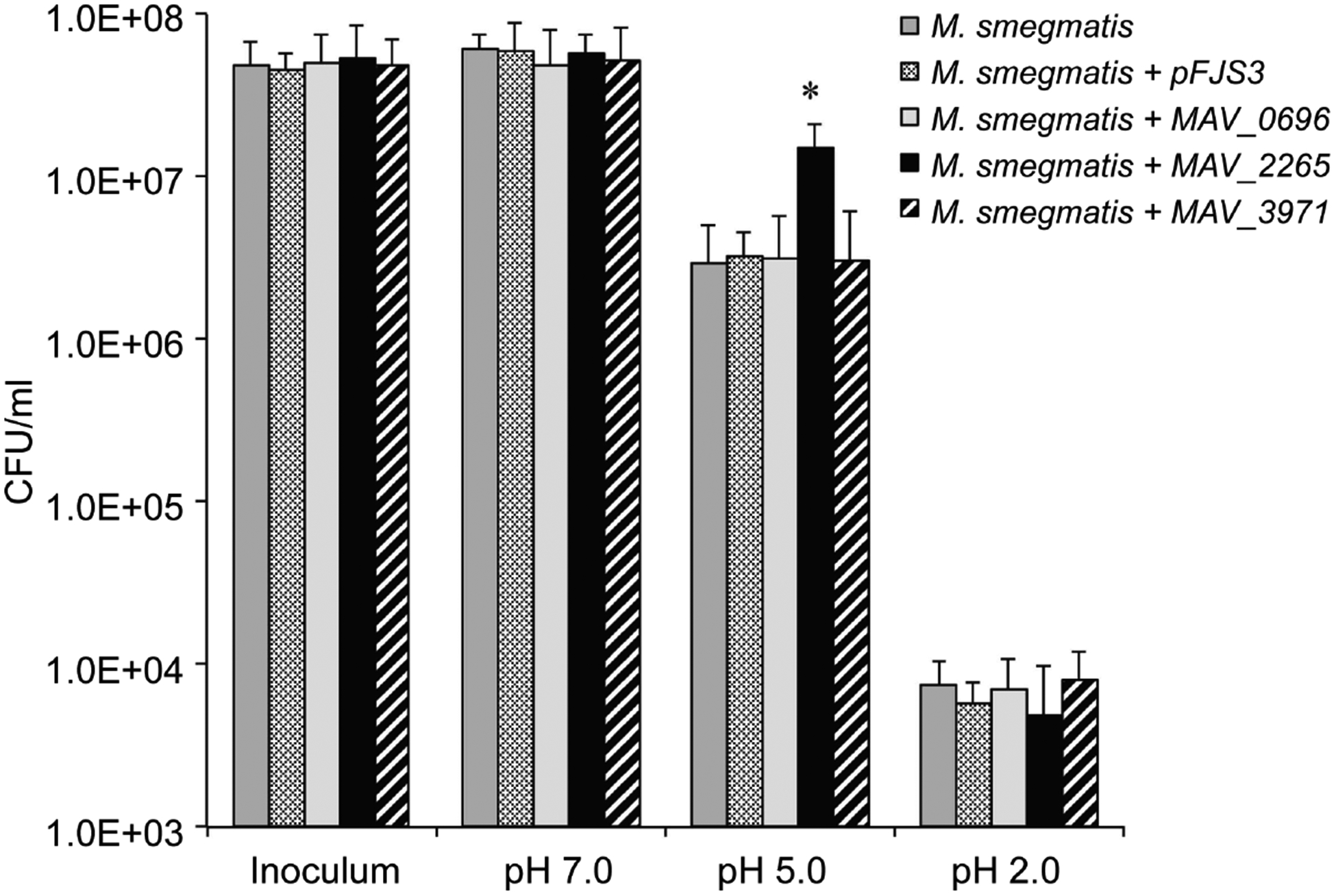
Viability of *M. smegmatis* prophage clones at different pHs: pH 2.0, pH 5.0 and pH 7.0. Bacteria were exposed to a range of different pH as described in Material and Methods. At pH 5.0, *M. smegmatis* overexpressing MAP_2265 was observed to have increased resistance to the environmental conditions. (*) p < 0.05 compared with the other genes.

Because *M. avium* may encounter acid in both the outside environment and when ingested by the host, we examined the effects of an acidic pH on bacterial growth (survival). *M. smegmatis* wild-type and prophage genes expressing clones from a five-day-old culture were resuspended in HBSS at acid pH 2.0, pH 5.0 and pH 7.0. Following 2 hours of exposure the acid pH was neutralized and the viability of *M. smegmatis*was determined by plating onto 7H10 plates. The average CFU is shown in Figure 3. Incubation of experimental and control strains of *M. smegmatis*in the neutral and highest acidic conditions (pH 2.0) did not result in any significant phenotype characteristic. However, MAV_2265 prophage gene containing *M. smegmatis* (1.5 × 10^7^) had significantly greater survival rate at pH 5.0 than both the wild-type control (2.9 × 10^6^) and the *M. smegmatis* containing control pFJS3 plasmid (3.2 × 10^6^). The presence of the other of prophages did not increase in bacterial resistance to acidic conditions (Figure 3).

### 3.5. Effects of UV Exposure on *M. smegmatis* Survival

**Figure 4.**
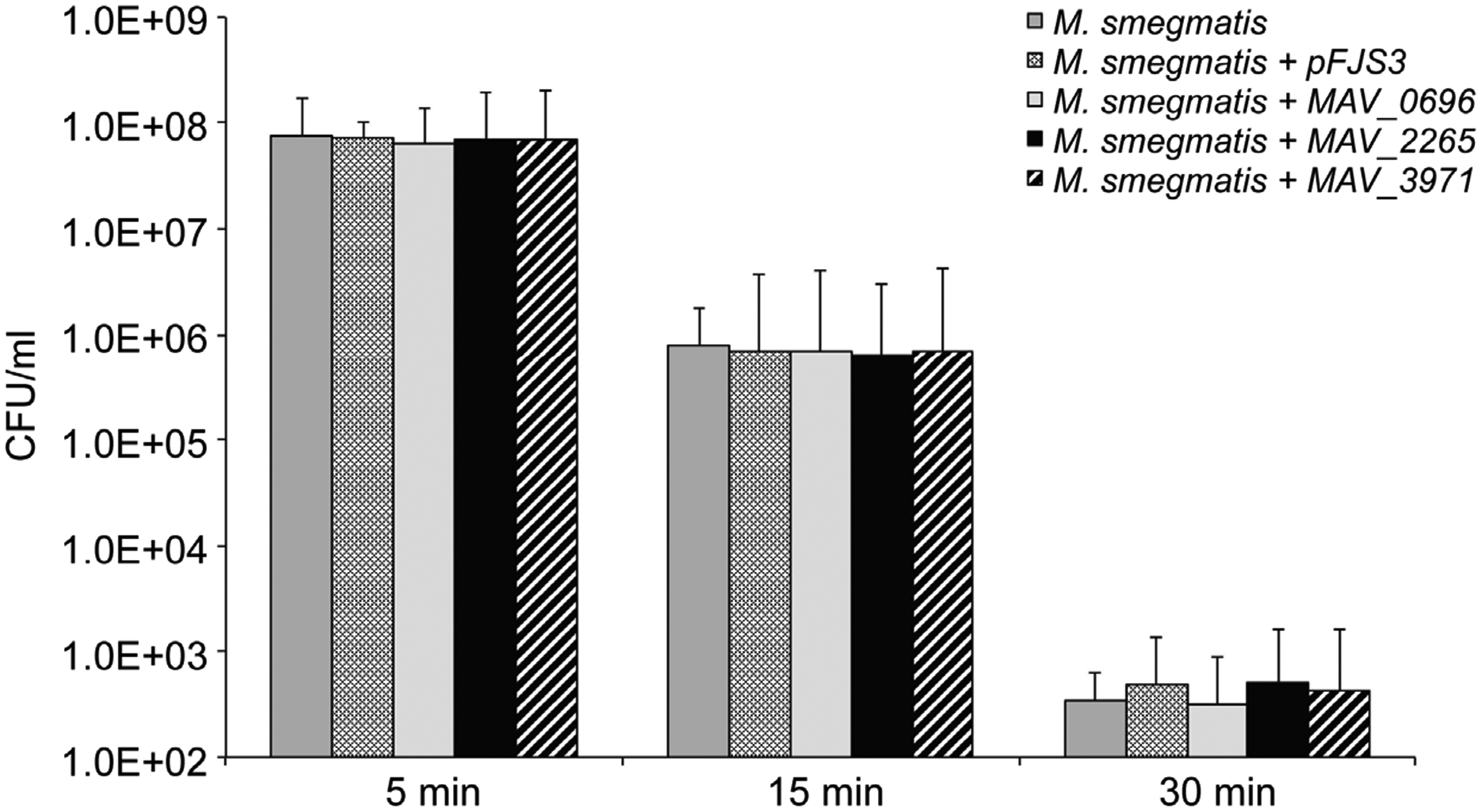
Viability of *M. smegmatis* prophage clones after 5 min, 15 min and 30 min exposure to UV light. UV light is an important environmental factor. The fact that overexpression of prophages had no effect on the clones to resist to UV light suggest that the phages are not associated with environmental protection.

*M. avium* is subjected to UV exposure from the sunlight in the environment. To evaluate whether prophage genes play a role in UV protection, we exposed the transformants and wild-type strains to UV light at different time intervals and compared the bacterial CFU before and after exposure. It was observed that while 15-min UV exposure resulted in 2-log reduction among transformants clones and wild-type strains of *M. smegmatis*, 30-min UV exposure led to over 5-log decrease in bacterial CFUs as shown in Figure 4. However, there was not any significance observed in survival rate of *M. smegmatis* clones expressing prophage genes compared to both control strains.

### 3.6. Expression of Prophages under Biofilm Formation

**Figure 5.**
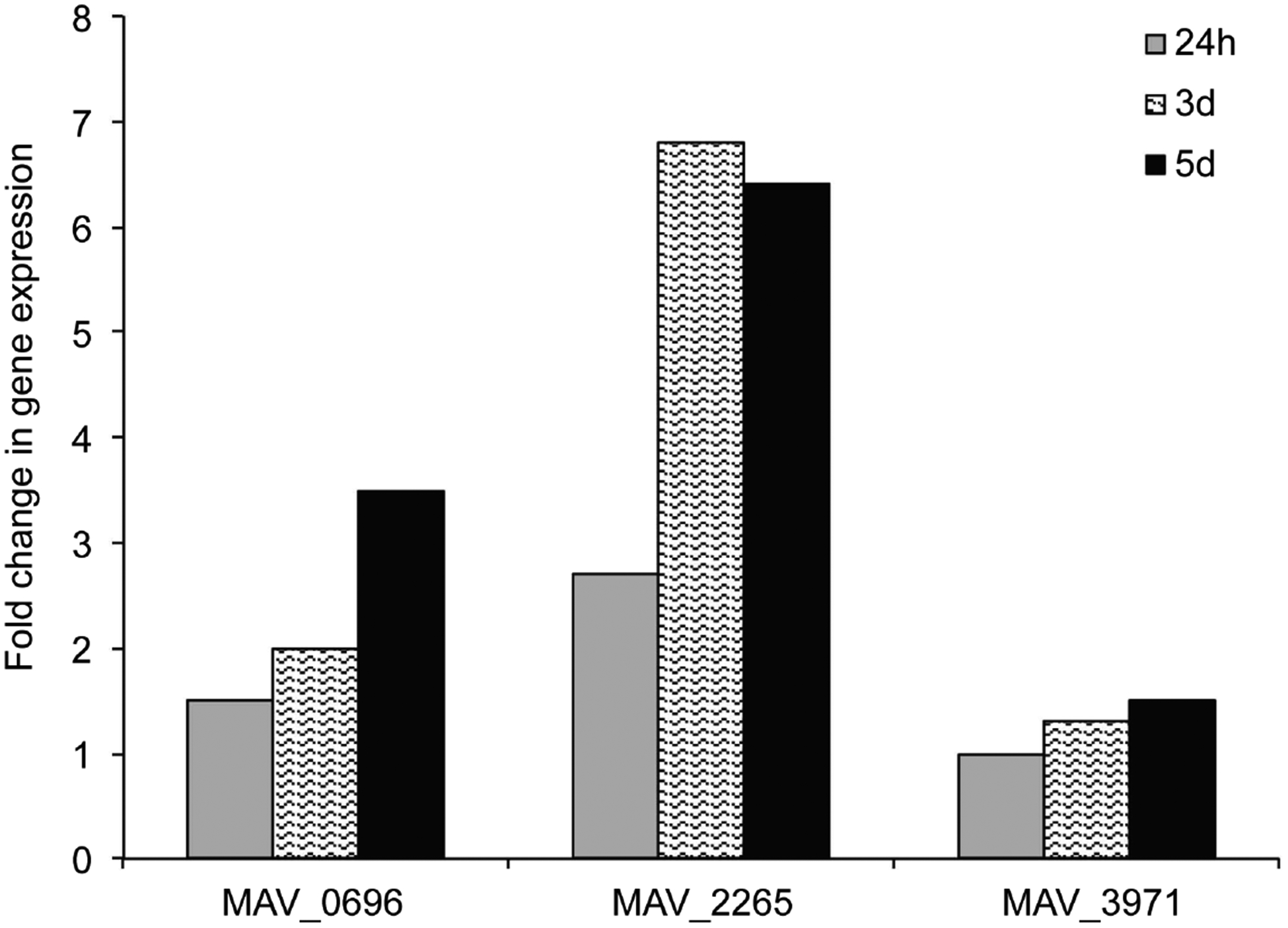
The Real Time PCR quantification of *M. avium* genes in biofilm. Bacterial RNA was purified, as described in materials and methods. Total RNA was used to determine the copy number of cDNAs for target prophage and reference 16S genes. Data were analyzed on the basis of Ct values of each sample and normalized with an internal housekeeping gene control, 16S rRNA. Values shown are representative of three different experiments with very similar results.

To examine whether prophage genes were upregulated upon biofilm formation, we performed the quantitative Real-Time PCR using *M. avium* RNA. The expression levels of target genes from biofilms and from broth-grown bacteria were normalized to the expression level of the endogenous reference 16S rRNA in each sample. The *M. avium* prophage gene MAV_2265 showed the greatest level of expression in biofilm at all selected time points compared with the expression levels of MAV_0696 and MAV_3971 (Figure 5). While MAV_0696 was upregulated over 2-fold at day 3, the induction increased up to 3.5 at day 5. There were not any changes observed in MAV_3971 gene expression levels over time as shown in Figure 5.

### 3.7. Survival in Macrophages

*M. smegmatis* expressing *M. avium* prophages sequences were used to infect THP-1 macrophages. *M. smegmatis* with the empty plasmid was used as control. None of the prophages sequences had any impact on the ability of *M. smegmatis*to survive in macrophages. By day 2 after infection, there was a reduction of 36%, 40%, 38%, and 39% for the *M. smegmatis* with the empty plasmid and expressing MAV_2265, MAV_0696 and MAV_3971 respectively. By day 3 after infection, the bacterial load reduction was 67%, 71%, 74%, and 68% respectively.

## 4. Discussion

Both humans and animals are susceptible to *M. avium* infection. This pathogen has the ability to form biofilm or a biofilm-like structure, which has been associated with chronic bacterial infection [16] [17]. Study of *M. avium* infection in the lung has suggested a possible association between biofilm formation and the difficulty in responding to therapy [19] [20]. In the experiments reported here, we attempted to determine whether chromosomal prophage genes had any role in the adaptation of *M. avium* to the environment. We screened each prophage gene transformant in *M. smegmatis* (gain of function assays), and compared the results with wild-type strains of *M. avium* 104 and *M. smegmatis*. The biofilm assay revealed that both MAV_2265 and MAV_0696 transformed into *M. smegmatis* resulted in a significant increase in biofilm formation at each indicated time interval, when compared to other strains used in this study. The degree of biofilm formation induced in *M*. *smegmatis* by the phage was even greater than the ability of *M. avium* 104 to form biofilm, which suggested that the prophage gene may act in synergism with other biofilm related genes. In addition, it is more likely that either biofilm or biofilm-like structure establishment is more regulated in *M*. *avium* 104 than in *M. smegmatis*, since wild-type *M. smegmatis* and all *M. smegmatis* transformants form more biofilm than *M. avium*.

A number of different prophages genes have been shown to be associated with virulence in bacteria such as *Staphylococcus aureus* [21] [22], *Streptococcus sp*. [22] [23] [24] and *Vibrio cholerae* [9] [25], *Shigella sp*. [26], *Pseudomonas aeruginosa* [27], *Salmonella enterica* [28], and others. In *S. aureus* for instance, prophages are expressed during animal infection and the absence of these phage genes results in virulence defects in a murine model of abscess formation [11], thus revealing essential contributions of prophages to the pathogenesis. Cholera toxin (ctx AB) is encoded by genes present in the prophage ctx0 [9] and is not produced in absence of the prophage. Prophages in *Streptococcus* and *Staphylococcus* are usually observed in “prophage regions” in the genome, in contrast to prophages in low CG content bacteria, such as gram-negative bacteria [29].

In the case of *M. avium*, we hypothesized that prophages would probably be linked to the ability of bacteria to interact with the environment. In fact, MAV_2265 and MAV_0696 were the prophage genes associated with the ability to form biofilm, and *M. smegmatis* MAV_2265 prophage clone had significant survival rate at pH 5.0 compared with control wild-type and other prophage clones. The MAV_2265 gene was upregulated in *M. avium* under conditions of biofilm formation by 6.8-fold at day 3. The expression of MAV_0696 resulted in a 3.5-fold increase in *M. avium* biofilms at day 5, while the evaluated MAV_3971 prophage gene showed no changes in expression over time.

The sequences of the three *M. avium* prophages in this study belong to XRE-family and DOC family. Two out of three sequences suggest regulatory function, which would explain the fact that they are located in isolated regions of the genome surrounded by transposases, phage intergrases and tRNA. Recently discovered prophages in *Salmonella* are linked to type III secretion systems such as SopE2 [30], demonstrating that prophage sequences can carry an array of functions associated with crucial phenotypes for pathogenic activity. Also recently, other prophages regions have been discovered in the *M. avium* genome. Since this finding happened after this paper had been submitted, the regions were not investigated.

In the case of MAV_2265, the discovery that the prophage gene is associated with an increase of biofilm formation raises important questions. The prophage GC content suggests acquisition of these genes from a different bacterial species, perhaps another environmental mycobacteria. The mechanism(s) involved of MAV_2265 and MAV_0696 participation in biofilm formation is still unknown at this point. Interestingly, the other prophage gene MAV_3971 has no significant role in any of the characteristics tested (acid resistance, temperature tolerance, biofilm formation, resistance to UV light), indicating that its function may be related with other aspects in the bacterial physiology. MAV_0696 is flanked by MAV_0697, which encodes for a protein containing a DNA metabolism/replication domain and may have a function associated with DNA replication under different stresses.

We have recently finished the sequence of 5 different *M. avium-M. intracellulare* complex strains (MAC 100, MAC 101, MAC A5, MAC 3388, MAC 3387). Search in the genome of the strains demonstrates that MAC 104 and MAC 101, two *M. avium* subsp. *hominissuis*, contain the MAV_0696 and MAV_2264 genes when their sequences are not present in the other strains. MAV_3971 in contrast is present in all strains. None of the sequences is present in *Mycobacterium tuberculosis, Mycobacterium marinum, Mycobacterium leprae*, which indicates that *M. avium* acquires them in a unique environment.

In summary, biofilm development has been shown to have an important impact on resistance to a number of stresses, including antibiotic exposure, while also having a significant impact on growth. Here we have identified three unique prophage sequences in the *M. avium* 104 genome and established that MAV_2265 and MAV_0697 genes participate in biofilm formation and low pH adaptation.

## Figures and Tables

**Table 1.** Strains used in this study.

Strains/plasmid	Purpose(s)
*M. avium* 104	Wild-type strain. Host for prophage gene amplification.
*M. smegmatis me*^2^ 155	Wild-type strain used as a host for *M. avium* prophage gene expression. Baseline control for all performed experiments.
pFJS3	Promoterless pMV161; Used for prophage gene construction.
*E. col* DH5B	Host for plasmid manipulation and propagation.

**Table 2.** The PCR primer sets for *M. avium* prophages containing genes.

Gene	Region	Primer sequence (5’−3’)	Amplicon size
MAV_0696	673526–673873	F-TTTTAAGCTT GCTTTGCGGCCATCCCT R-TTTTTAAG CTTTTAGG CGGT GGTGACC	347 bp
MAV_2265	2269684–2270418	F-TTTTTAAGCTCGGTACCCCGGTCCG R-TTTTTAAGCTTTTACGTTTCTGGGTCA	735 bp
MAV_3971	4097430–4097943	F-TTTTTAAGCTTCGATGCAGGCTGTTGC R-TTTTTAAG CTTTCAGTCCTCTTGGTAG	534 bp

**Table 3.** Putative prophage genes found in the genome of *M. avium* 104.

Gene	GC content	Product Description/Conserved Domains
MAV_3971		Death-on-curing protein. Contains Fic (filamentous induced by cAMP) domain. 50% identical to the C-terminus of Gp30 protein of Mycobacterium phage Giles
MAV_0696	59%	Hypothetic protein. Contains DNA-binding helix-turn-helix domain.
MAV_1165	64%	Putative prophage regulatory protein. The DNA-binding helix-turn-helix XRE-family like protein. Belongs to the xenobiotic response element family of transcriptional regulators.

**Table 4.** Growth rate in 7H9 broth of *M. smegmatis* WT compared to *M. smegmatis* clones expressing MAC_2266, MAC_0691 and MAC_3971.

	Growth		
Strains	Baseline	3 days	7 days
*M. smegmatis* WT (PMV161)	3.0 ± 0.4 × 10^4^	8.1 ± 0.5 × 10^4^	1.4 ± 0.6 × 10^5^
*M. smegmatis*. MAC_1165	1.5 ± 0.3 × 10^4^	6.9 ± 0.3 × 10^4^	1.0 ± 0.3 × 10^5^
*M. smegmatis*. MAC_0691	1.8 ± 0.1 × 10^4^	7.1 ± 0.4 × 10^4^	1.1 ± 0.3 × 10^5^
*M. smegmatis*. MAC_3971	1.9 ± 0.5 × 10^4^	7.8 ± 0.3 × 10^4^	9.8 ± 0.5 × 10^5^

**Table 5.** Biofilm formation by *M. smegmatis* clones expressing the prophage containing genes.

Bacteria	Absorbance/550 nm			
	3 days	5 days	7 days	14 days
*M. avium* 104	1.107 ± 0.432	3.604 ± 0.475*	4.926 ± 0.438*	7.586 ± 0.365*
*M. smegmatis*	0.478 ± 0.263	1.438 ± 0.251	2.481 ± 0.195	3.671 ± 0.402
*M. smegmatis* + pFJS3	0.524 ± 0.058	1.387 ± 0.135	2.516 ± 0.362	3.636 ± 0.356
*M. smegmatis* + MAV_0696	1.618 ± 0.355*	4.156 ± 0.391*	4.881 ± 0.453*	5.357 ± 0.218*
*M. smegmatis* + MAV_2265	1.336 ± 0.160*	3.952 ± 0.333*	4.216 ± 0.274*	6.417 ± 0.306*
*M. smegmatis* + MAV_3971	1.022 ± 0.276	1.057 ± 0.381	2.543 ± 0.166	3.578 ± 0.247

* p < 0.05 compared with *M. smegmatis* wild-type and *M. smegmatis* + pFJS3. 3 different clones expressing the genes were analyzed. All three clones expressing the same gene showed similar results. The mean + SD of one clone is shown.
